# Multi-Tissue Transcriptome Study of Innate Immune Gene Expression Profiling Reveals Negative Energy Balance Altered the Defense and Promoted System Inflammation of Dairy Cows

**DOI:** 10.3390/vetsci10020107

**Published:** 2023-02-01

**Authors:** Lingli Dai, Zaixia Liu, Lili Guo, Yuan Chai, Yanda Yang, Yu Wang, Yanfen Ma, Caixia Shi, Wenguang Zhang

**Affiliations:** 1 College of Animal Science, Inner Mongolia Agricultural University, Hohhot 010018, China; 2 Veterinary Research Institute, Inner Mongolia Academy of Agricultural and Animal Husbandry Sciences, Hohhot 010031, China; 3 College of Veterinary Medicine, Inner Mongolia Agricultural University, Hohhot 010018, China; 4 School of Agriculture, Ningxia University, Yinchuan 750021, China; 5 Inner Mongolia Engineering Research Center of Genomic Big Data for Agriculture, Hohhot 010018, China; 6 College of Life Science, Inner Mongolia Agricultural University, Hohhot 010018, China

**Keywords:** negative energy balance, innate immune, defense, inflammation, GSEA

## Abstract

**Simple Summary:**

The perinatal period is very important for dairy cows. Dairy cows have higher energy and nutrient requirements during pregnancy and lactation than they do during the rest of their lives because of physiological changes like fast growth and uterine development. As a result, high-yielding dairy cows frequently experience severe negative energy balance (NEB) during the perinatal period. This NEB causes metabolic and immunological abnormalities in dairy cows, which in turn causes systemic inflammation and reduced resilience. In this study, transcriptome data from multiple tissues were utilized to analyze the impacts of NEB on innate immune genes, as well as the mechanism underlying the alteration of the ’host defense and systemic inflammation. According to studies, NEB downregulates the defensive function of innate immune genes in the endometrium, resulting in lower defense capability, upregulation of defense and inflammatory responses in other tissues, activation of systemic defense responses, and promotion of systemic inflammation. These findings provide crucial clues for the diagnosis and prevention of systemic inflammation brought upon by perinatal NEB.

**Abstract:**

Negative energy balance (NEB) during the perinatal period leads to metabolic and immunological disorders in dairy cows, resulting in systemic responses and inflammation. The innate immune system is crucial for the host’s protection and inflammatory response. However, systematic research is still lacking on how NEB affects the innate immune system to alter the ’host defense capability and inflammatory response. In this investigation, raw transcriptome data of adipose, blood, endometrial, hypothalamus, and liver tissues were downloaded from a public database, cleaned, aligned, quantified, and batch-corrected. The innate immune gene list was retrieved from innateDB, followed by the expression matrix of innate immune genes in various tissues for differential expression analysis, principle component analysis (PCA), and gene set enrichment analysis (GSEA). Under the effect of NEB, adipose tissue had the most differentially expressed genes, which were predominantly up-regulated, whereas blood GSEA had the most enriched biological processes, which were predominantly down-regulated. The gene sets shared by different tissues, which are predominantly involved in biological processes associated with defense responses and inflammation, were dramatically down-regulated in endometrial tissues and highly up-regulated in other tissues. Under the impact of NEB, LBP, PTX3, S100A12, and LCN2 play essential roles in metabolism and immunological control. In conclusion, NEB can downregulate the defensive response of innate immune genes in endometrial, upregulate the immune and inflammatory response of other tissues, activate the host defense response, and increase the systemic inflammatory response. The analysis of the effects of NEB on innate immune genes from the multiple tissues analysis provides new insights into the crosstalk between metabolism and immunity and also provides potential molecular targets for disease diagnosis and disease resistance breeding in dairy cows.

## 1. Introduction

The perinatal period is one of the most crucial physiological stages for dairy cows. Due to physiological changes such as rapid fetal growth, parturition, and lactation, the demand for energy and nutrition increases while the appetite and feed intake of dairy cows decrease. This is why dairy cows generally experience different levels of negative energy balance (NEB) during the perinatal period, with high-yielding dairy cows frequently experiencing severe negative energy balance [1,2]. Despite the action of homeostatic mechanisms to maintain blood parameters within physiologic levels, changes in metabolites and hormones occur as a result of increased metabolic demands in lactating animals. These changes are not necessarily indicative of diseases but make animals physiologically unstable and more susceptible to a number of metabolic diseases at this stage than during other life periods compromising productivity [3,4]. In particular, dairy cattle are predisposed to sickness at any time, especially after calving and during early lactation [5]. Due to energy shortage, the host generates non-esterified fatty acids (NEFA) by adipose mobilization, which enter the liver through the blood to produce ketone body β-hydroxybutyric acid (BHBA) and are delivered to peripheral tissues with blood circulation to provide energy. If cows fail to adapt to such metabolic changes, severe NEB will lead to continuous blood accumulation of NEFA and BHBA, which will eventually evolve into ketosis [6] and affect the production and reproductive efficiency of cows [7,8].

NEB alters the normal physiological function of multiple tissues in dairy cows. NEB causes fat mobilization, and excessive lipolysis triggers an inflammatory response in adipose tissue. The accumulation of NEFA and BHBA in the blood causes immunosuppression of immune cells [9]. In difficult situations, NEB can lead to abnormal liver metabolism, increased oxidative stress, increased release of proinflammatory cytokines, and liver damage [10]. Cows with severe NEB are prone to endometriosis and endometritis after delivery due to obstructed endometrial repair [11,12,13]. Because of inflammation in the hypothalamus, cows consume less feed during NEB. This is because hypothalamic inflammation affects hypothalamic orexigenic and anorexigenic signaling in early lactation [14].

Additionally, aberrant hypothalamic activity influences neuropeptide release, which in turn modulates food intake and energy expenditure, which ultimately results in systemic reactions [15]. NEB is responsible for metabolic and immunological problems in dairy cows.

Severe NEB produces immunosuppression, which is frequently accompanied by systemic inflammation and makes individuals susceptible to a variety of infectious illnesses, including endometritis and mastitis [16]. Innate immunity plays an important role in the development of inflammation [17]. As the first line of immune defense, innate immunity plays an important role in resisting microbial invasion and maintaining homeostasis. The metabolic imbalance caused by NEB leads to immune disorders, which in turn further aggravate the metabolic disorders [17,18,19]. However, there is still a lack of systematic research on how innate immunity is affected during this process. In this study, we analyzed the effects of NEB on innate immune genes in adipose, blood, endometrium, hypothalamus, and liver through transcriptome data and systematically investigated the effects of NEB on innate immune genes on defense and systemic inflammation in dairy cows, in order to provide new insights for the study of the interaction mechanism between metabolism and immunity, and lay the foundation for the molecular diagnosis and disease resistance breeding of dairy cows.

## 2. Materials and Methods

### 2.1. RNA Sequencing Data and Data Grouping Information

- RNA Sequencing Data Sources

The RNA-seq next-generation sequencing (NGS) data of negative energy balance and ketosis-associated transcriptome in dairy cows was obtained from a public database (GEO database). The original sequencing data for liver, adipose, hypothalamus, endometrial, and blood corresponded to the following GEO numbers: 24 adipose samples [20] (https://www.ncbi.nlm.nih.gov/geo/query/acc.cgi?acc=GSE159224; accessed on: 8 October 2020), 23 peripheral blood leukocyte samples [21] (PRJNA605719), 24 Endometrial samples [22] (https://www.ncbi.nlm.nih.gov/geo/query/acc.cgi?acc=GSE169638; accessed on: 25 March 2020), 21 hypothalamus samples [23] (https://www.ncbi.nlm.nih.gov/geo/query/acc.cgi?acc=GSE49540; accessed on: 5 August 2013), 18 liver samples [24] (https://www.ncbi.nlm.nih.gov/geo/query/acc.cgi?acc=GSE37544; accessed on: 24 April 2012). The raw fasta data from all the samples were used for the subsequent analysis.

- Data grouping information

According to the progression of NEB, we separated the data from multiple tissues into two broad groups: normal and NEB. (1) Samples of adipose were collected from the abdomen Subcutaneous AT of perinatal (preP) and postpartum (PP1) dairy cows. Subcutaneous AT samples were collected from Holstein cows (*n* = 12) at 11 ± 3.6 d before calving date (PreP) and at 6 ± 1 d (PP1) after parturition. Significant postpartum blood ketone body, BHBA, and NEFA concentrations increased dramatically (BHBA from 6 mg/dL to 9 mg/dL on average, NEFA from 0.1 mEq/L to 0.45 mEq/L on average), whereas blood glucose declined significantly, indicating that cows had significant postpartum NEB. Accordingly, the preP adipose transcriptome data were separated into the normal group, and the postpartum (PP1) adipose sample data were separated into the NEB group. (2) Blood leukocyte transcriptome samples were collected from healthy cows, ketosis and subclinical ketosis cows, and sample data from the healthy cow (blood BHBA < 1.4 mmol/L) was classified as the normal group, whereas sample data from the ketosis and subclinical ketosis cow (blood BHBA ≥ 1.4 mmol/L) was classified as the NEB group. (3) Cows with mild NEB (MNEB) and severe NEB (SNEB) provided the endometrial samples, and the difference between the two sets of samples was the progression of NEB. Therefore, the data for MNEB cows were separated into the generally normal group, and the data for SNEB cows were separated into the NEB group. (4) The hypothalami of cows with an NEB condition caused by an 18-day dietary restriction model were collected. In this study, the data from the group treated with dietary restriction belonged to the NEB group, while the data from the control group with a higher level of feeding belonged to the normal group. (5) The liver samples were from MNEB cows which were fed ad libitum grass silage and 8 kg/day concentrates and milked once daily) and SNEB cows which were fed 25 kg/day silage and 4 kg/day concentrate and milked three times daily. Relative to MNEB cows, SNEB cows had undergone significant (*p* < 0.05) changes in energy balance (−3.6-fold), blood glucose (−1.5-fold), blood NEFA (+4.7-fold), blood BHBA (+7.4-fold), Similar to endometrial data, liver transcriptome data were grouped into MNEB and normal subgroups, and SNEB and NEB subgroups. The detailed information is shown in the following table (Table 1).

All the above data were taken from research examining ketosis or negative energy balance in dairy cows. Samples of adipose tissue and blood leukocytes were taken from prenatal and postpartum cows, respectively. The blood concentrations of BHBA and NEFA increased considerably, and the cows suffered NEB. The samples of endometrial tissue, hypothalamus tissue, and liver tissue used in this study came from cows that had negative energy balances following dietary energy restriction. All of the involved research focused on the influence of negative energy balance on the physiological condition of cows. Following the abovementioned classification, the data were analyzed as follows.

### 2.2. Reads Mapping and Quantification of Gene Expression

Fastp software [25] was used to filter the raw data (reads), yielding high-quality clean reads. Reads with linkers are deleted; reads with N (meaning the base information could not be determined) content greater than 10% are deleted; low-quality reads are deleted (reads with base quality less than 3 or average base quality less than 15). For the following analysis, only clean reads were used.

The bovine reference genome ARS-UCD1.2 [26] was obtained from the Ensembl genome browser (https://www.ensembl.org; accessed on: 1 March 2021) [27] and HISAT2 ver. 2.1.0 (Johns Hopkins University Center for Computational Biology, Baltimore, MD, USA) [28] was used to provide a reference genome index for the alignment. The clean readings were mapped to the reference genome before being assembled and quantified as transcripts. StringTie [29] was used for transcript construction and quantification. The total gene count list of the five tissues was de-batch corrected using the combat_seq command from the ‘sva’ [30] R package. Using the ‘GenomicFeature’ [31] R package, the TPM value of the corrected gene count list was calculated, obtaining the TPM matrix of five tissue gene expression levels. Subsequently, we performed differential gene expression analysis between NEB group versus normal group in every tissue using ‘DESeq2’ package [32] in R.

### 2.3. Innate Immune Gene Sets and Principal Component Analysis

We used OrthoFinder [33] to analyze and compare human innate immune gene data collected from the InnateDB database (https://www.innate.ca; accessed on: 15 May 2008) to discover bovine innate immune genes, and when combined with the bovine innate immune gene set from the InnateDB database, resulting in a total of 1232 bovine innate immune genes.

The above 1232 bovine innate immune genes were matched with gene expression levels in different tissues to generate a TPM data list of the expression levels of bovine innate immune genes in different tissues. Principal component analysis (PCA) was performed by using basic PCA tools in Hiplot (https://hiplot.org; accessed on: 23 September 2022), a comprehensive and easy-to-use web service for boosting publication-ready biomedical data visualization [34]. The ‘heatmap’ R package [35] was used to generate the gene expression heatmap in order to examine the expression of innate immune genes in tissues with negative energy balance.

### 2.4. Gene-Set Enrichment Analysis and Network Architecture

The list of innate immune gene expression in each tissue was analyzed using gene-set enrichment analysis (GSEA) [36] and compared between the NEB and normal groups. The GSEA software version 4.1.0 (The University of California San Diego, Baltimore, MD, USA) was utilized, and the c5.go.bp.v7.2.symbols database was employed. We used the obtained *p*-values to perform a GSEA based on the weighted Kolmogorov–Smirnov Test (WKS). The gene sets with *p*-values less than 0.05 were selected and imported into the Cytoscape [37,38] software for visualization. Using the merge method, biological processes enriched in different tissues were then clustered according to similarity. The shared genes associated with defense were entered into a string database to generate a protein–protein interaction (PPI) network, which was then loaded into the Cytoscape application for visual analysis.

## 3. Results

### 3.1. Expression of Innate Immune Genes in Different Tissues of Dairy Cows

**Figure 1:**
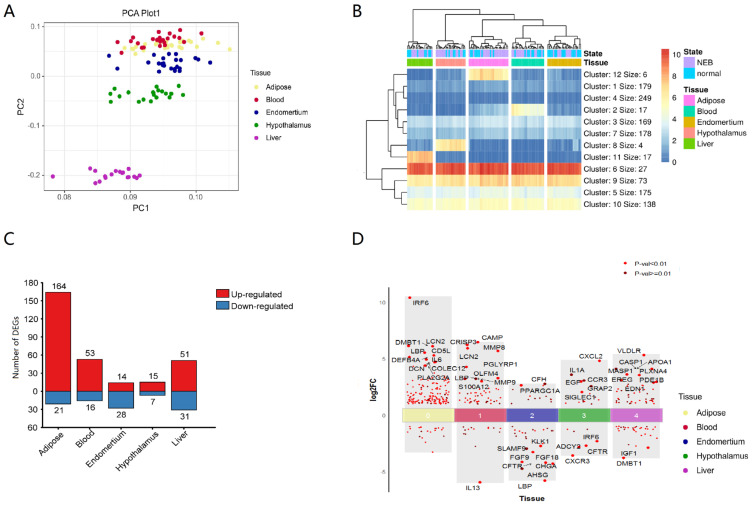
Transcriptional expression analysis of innate immune genes in different tissues. (**A**) PCA of 1232 bovine innate immune genes from different tissues. (**B**) Heatmap of the transcriptional expression of innate immune genes from different tissues, Kmer = 12, (**C**) DEGs (Log2FC > 1, *p*-value < 0.05) of innate immune genes from different tissues. (**D**) TOP 10 innate immune DEGs of different tissues.

To investigate global biological alteration and systemic whole-body impacts of innate immune genes related to NEB, we obtained the transcriptome data of adipose, blood, endometrial, hypothalamus, and liver tissue from a public database of dairy cows. The reads counts matrix of expressed genes from 141 samples was transformed into a TPM (Transcripts Per Million) matrix. The TPM of innate immune genes exhibited a closed grouping of adipose, blood, and endometrial samples in principal component analysis (PCA). Liver and hypothalamus samples distinguished out from other tissues (Figure 1A). This suggested that innate immune genes in different tissues had distinct patterns.

To confirm whether there were genes with specifically high expression in different tissues, a heatmap of gene expression was generated, the Minkowski distance between genes and samples was computed, and k-means clustering was done. The K value of 12 distinguishes each tissue. The other four tissues expressed innate immune genes at high levels except for endometrium (Figure 1B, Table S1). Innate immune genes were substantially expressed in some organs. The function of these genes was closely related to the function of tissues and organs. In blood, triggering receptor expressed on myeloid cells 1 (TREML1) [39,40], defensin beta 4 (DEFB4A) [41], fatty acid receptor 2 (FFAR2) [42], and interferon-stimulated exonuclease 20 (ISG20) [43] genes were greater, which affected blood cell immunity and metabolism. In the liver, genes associated to complement (C5, C6, C8A, C8B, C9, C4BPA) and forkhead transcription factors (FOXA1, FOXA2) were substantially elevated. The liver was a significant source of complement [44], and hence the high expression of complement-related genes was consistent with the physiological function of the liver. Forkhead transcription factors [45] control metabolism genes and are crucial to liver metabolism. Calcium/calmodulin-dependent protein kinase IIα (CAMK2A), chromogranin A (CHGA), arginine vasopressin (AVP), and atypical Notch ligand (DLK1) were abundantly expressed in the hypothalamus, and involved in signal transduction, hormone secretion, and other functions [46,47,48,49,50]. In adipose tissue, five genes, including transcription factors HOXA9, ADIPOQ, LEP, and CD209, were substantially expressed, which are connected to the maintenance of adipose tissue function and differentiation [51,52,53]. Thus, tissue-specific functions may be related to innate immune gene expression patterns.

The number of differentially expressed innate immune genes in different tissues affected by NEB was also different. Adipose tissue had the most DEGs and differentially expressed innate immune genes, whereas the hypothalamus had the least (Figure 1C), showing that NEB had the greatest effect on gene expression in adipose tissue. The differentially expressed innate immune genes were largely up-regulated in adipose, blood, and liver tissues and down-regulated in endometrial tissues (Figure 1D).

All of the top 10 DEGs of innate immune genes (IRF6, DMBT1, LCN2, LBP, CD5L, DEFB4A, IL-6, COLEC12, DCN, PLA2G2A) that were significantly differentially expressed in adipose tissue were up-regulated, with IRF6, LCN2, LBP, DEFB4A, and IL-6 also being differentially expressed in other tissues (Table S2). Nine genes (CAMP, CRISP3, MMP8, LCN2, PGLYRP1, OLFM4, MMP9, LBP, S100A12) were increased among the 10 most substantially differently expressed innate immune genes in the blood NEB group, while only IL13 was down-regulated. Two genes (CFH, PPARGC1A) were up-regulated, and eight were down-regulated in the NEB group of the endometrium (LBP, AHSG, CFTR, CHGA, FGF9, FGF18, SLAMF9, KLK1). Six genes (CXCL2, IL1A, CCR3, EGF, GRAP2, SIGLEC1) were increased in the hypothalamus, whereas four genes were down-regulated (CXCR3, CFTR, ADCY2, IRF6). Eight genes (VLDLR, APOA1, CASP1, MASP1, PLXNA4, EREG, PDE1B, and EDN1) were up-regulated in the liver, whereas two genes were down-regulated (DMBT1, IGF1).

### 3.2. Functional Analysis Reveals Widespread Alterations of Biological Processes of Innate Immune Gene Post NEB Condition with Different Tissue

**Figure 2:**
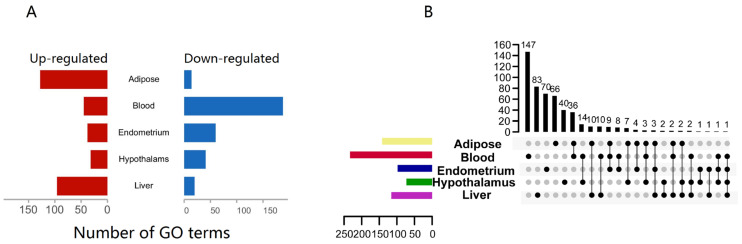
GSEA of innate immune genes from different tissues. (**A**) Number of GO(BP) terms enriched by GSEA (*p*-value < 0.05); (**B**) upset plot of the GO(BP) terms enriched by GSEA (*p*-value < 0.05), both the x-axis and y-axis for the numbers of terms.

We utilized gene-set enrichment analysis (GSEA) with gene ontology (GO) biological processes to uncover NEB-impacted biological functions (BPs). We clustered GO terms with *p*-values below 0.05 when comparing NEB to normal in different tissues. Most pathways, 233, were enriched in the blood and mostly down-regulated, indicating that NEB inhibited blood innate immune gene activation (Figure 2A). Adipose and hepatic routes predominated over the hypothalamus and endometrial pathways. Few pathways were shared by several tissues, and most of the pathways enriched to innate immune genes were tissue-specific (Figure 2B).

**Figure 3:**
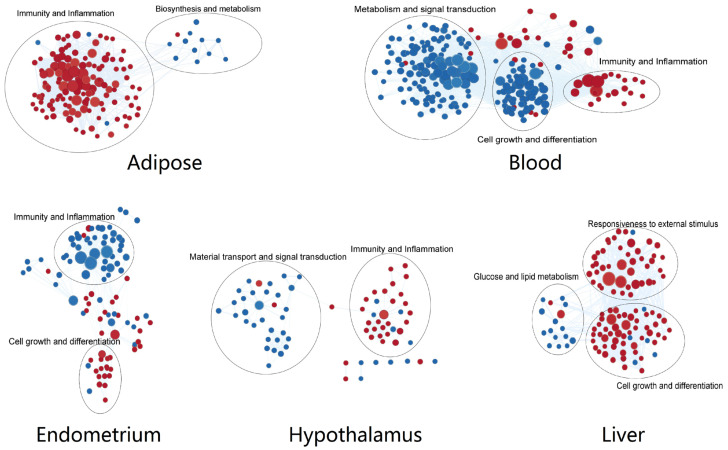
Cluster of the GO(BP) terms from every tissue. Red dots for up-regulated processes in NEB group and blue dots for down-regulated ones.

The pathways enriched in different tissues were clustered according to similarity co-efficient (Figure 3). Processes related to immune response and inflammation were significantly up-regulated in the adipose and hypothalamus but down-regulated in the endometrium, related to defense reaction were up-regulated in the blood, and related to the response to the external stimulus were up-regulated in the liver. It was seen that pathways related to defense function were generally up-regulated and influenced by NEB, except in the endometrium, where they were mainly down-regulated.

We also observed that innate immune genes not only performed defense-related functions but were also involved in metabolic processes, signal transduction, and cell growth and differentiation. The pathways related to metabolism were significantly down-regulated by NEB in the adipose, blood, and liver. The pathways associated with signal transduction were mainly down-regulated in the blood and hypothalamus. The pathways associated with cell growth and differentiation were down-regulated in blood and the hypothalamus, with opposite trends in the liver.

These demonstrate that NEB extensively altered the defense, metabolic and signaling functions of innate immune genes.

### 3.3. Tissue-Specific Altered Biological Functions Point to Specificity of Defensive, Metabolic, and Signaling Responses to NEB

Next, we looked at the specificity of biological processes that were significantly affected in various tissues as a result of NEB. According to the normalized enrichment score (NES) (*p*-value = 0.05), the gene sets were ordered. We concentrated on the top five and bottom five most significant GO terms (Table 2).

The adipose tissue had the most changes in humoral immune response, including humoral immune response mediated by circulating immunoglobulins, humoral immune response regulation, and complement activation, all of which were increased. The metabolism of fatty acids and sterols was markedly suppressed in adipose tissue.

In the blood, leukocyte-mediated immune response-related pathways were significantly up-regulated, such as myeloid leukocyte-mediated immunity, exocytosis, myeloid leukocyte activation, and defense response to Gram-positive bacterium. However, pathways related to humoral immunity, such as regulation of immunoglobulin production, B cell activation involved in the immune response, and immunoglobulin production, were suppressed.

Positive regulation of Jun kinase activity, which plays an important role in immune response and metabolism, was down-regulated in the endometrium, while both a cellular response to the virus and positive regulation of response to cytokine stimulus pathways were suppressed. But lymphocyte chemotaxis was enhanced. The same with the adipose, ‘Sterol Metabolic Process’ was also down-regulated.

In the hypothalamus, processes related to the regulation of CD4-positive T-cell differentiation and leukocyte homeostasis were significantly up-regulated. The pathways associated with signal transduction were down-regulated, such as the regulation of calcium-ion transmembrane transport, calcium-ion transmembrane import into the cytosol, and the non-canonical WNT signaling pathway. Interestingly, the cellular response to ketone was down-regulated in the hypothalamus due to NEB.

We observed that tissue migration [54] was up-regulated, and the glucose metabolic process was down-regulated in the liver. We found that the functions of innate immune genes affected by NEB were not identical and were related to the functional properties of the tissue.

### 3.4. Network Analyses of Gene Sets from Multi-Tissue Unveils Universal Changes to the Defense Response Caused by NEB

**Figure 4:**
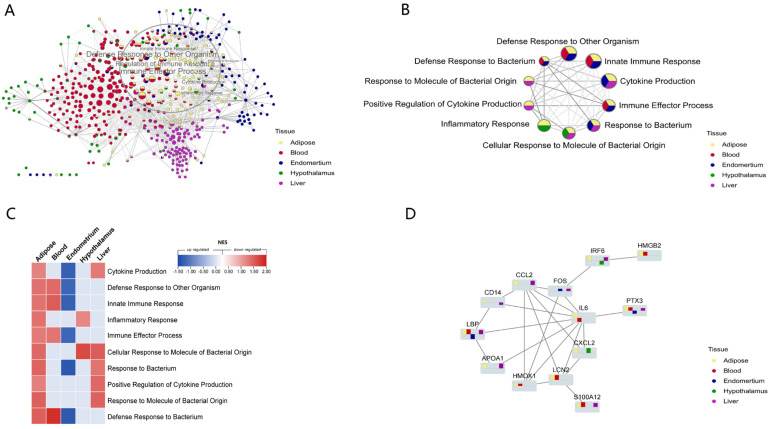
NEB altered the defense and inflammation-related processes and innate immune genes. (**A**) The cluster of the biological processes enriched from different tissues by GSEA as similarity, and different colors of pie plots means shared processes of multi-tissue. The processes in the circle are mainly related to defense and inflammation. (**B**) The network of shared hub processes related to defense and inflammation from multi-tissue. (**C**) The heatmap of NES value of the processes enriched from the five tissues. (**D**) The PPI network of the shared DEGs with great differences. The bar graph shows upregulation above the baseline and downregulation below. The y-axis shows Log2FC.

Then, using the Cytoscape ‘merge’ tool, we combined all of the networks of routes enriched from different tissues based on similarity coefficients. Overall, these pathways were grouped according to tissue specificity, indicating that innate immune gene expression patterns were tissue specific. However, there were several common defense-related pathways enriched from diverse tissues that were practically concentrated in the same location (Figure 4A). These multi-tissue common pathways established a highly linked defensive network, all of which were elevated in adipose tissue (Figure 4B,C). NEB caused excessive fat mobilization and increased inflammation in adipose tissue [20,55], as well as a change in the overall defense state. The network’s highest weighted variable was ‘Defense Response to Other Organism,’ which was significantly affected in three tissues (adipose, blood, and endometrium). This suggested that NEB modifies the host defense mechanisms against foreign substances and influences how innate immune genes are expressed. We discovered that several common processes, such as ’Cytokine Production‘, ’Defensive Response to Bacterium‘ and ’Innate Immune Response‘ were down-regulated in the endometrium, decreasing the endometrial defense capability (Figure 4C). Four of these pathways—innate immune response, reaction to bacteria, response to other organisms, and defense response to bacterium—were present in adipose, blood, and endometrial and were elevated in all three tissues while being down-regulated in the latter. We noted that an ‘Inflammatory response’ was promoted both in the adipose and hypothalamus.

There were 54 related DEGs across the processes that these tissues shared. These DEGs were added to the STRING database (https://cn.string-db.org/; accessed on: 12 August 2021) to create a PPI network, which was then laid out in Cytoscape using the yfiles Hierachic layout method. We discovered that the PPI network was centered on IL-6 (Figure 4D). CXCL2, LCN2, APOA1, LBP, CCL2, IRF6, HMGB2, PTX3, and S100A12 genes were significantly differentially expressed and interacted with IL-6 in different tissues. HMOX1, FOS, and CD14 also connect these proteins to form an interaction network (Figure 4D).

## 4. Discussion

Traditional dairy cow breeding focuses on increasing milk or protein output while ignoring functional qualities and health-related traits [56]. Long-term selection to boost production causes physiological and immunological imbalance, especially in the early lactation period, when cows have the most severe metabolic imbalance and immune imbalance, making them more susceptible to environmental influences and secondary illnesses [57]. Negative energy balance has a variety of negative effects on dairy cow health. There have been a lot of investigations on how NEB affects dairy cows’ metabolism, reproduction, lactation, and other physiological processes, but there has not been any comprehensive study of the cows’ innate immunity. With a focus on the influence of NEB on the expression of innate immune genes, the transcriptome data of the adipose, blood, endometrium, hypothalamus, and liver of dairy cows were analyzed, and the effect on the host was investigated.

The biological processes enriched by GSEA analysis of innate immune genes in various tissues revealed that blood, endometrial, and hypothalamus gene functions were primarily down-regulated owing to the NEB effect, while adipose and liver tissues were primarily up-regulated, primarily because adipose is the primary energy storage tissue and liver is the main metabolic tissue, and negative energy balance primarily produced abnormal metabolic activity in adipose and liver.

Innate immune genes are engaged not only in host defense, immunity, and inflammatory processes but also in biological processes influenced by negative energy balance, such as synthesis and metabolism, substance transport, and signal transduction. NEB increases the inflammatory and immunological responses of adipose, which, as an energy-consuming process, will aggravate the negative energy balance. During cows’ perinatal stage, innate immune gene-related metabolic and substance production activities, such as the fatty acid metabolic process and the sterol metabolic process, were repressed [58,59,60,61]. These biological processes were down-regulated in the NEB group, which appears to contradict the high energy demands of immunity and inflammation and the exacerbation of lipolysis through fat mobilization. This is due to the fact that our research concentrated on innate immune genes. Adipose tissue also possesses many non-innate immune genes that compensate for innate immune gene-induced glucose and lipid metabolic downregulation. In adipose tissue, innate immunity up-regulates inflammatory and immune-related pathways but also down-regulates glucose and lipid metabolism and steroid production and metabolism pathways to counteract excessive inflammation and immune response, which is also a balancing strategy for tissues to transition from an imbalanced to a balanced state.

In endometrial tissue, innate immune genes are also involved in cell proliferation, differentiation, protein synthesis, and secretion, and NEB has a significant impact on these processes. Cows with severe NEB (ketosis) are sensitive to endometritis, which has a significant impact on reproductive performance. The endometrium is a tissue susceptible to serious harm from pathogenic bacteria. In this analysis, NEB regulates innate immune responses in the endometrium, manifesting predominantly as immunological down-regulation of ‘Cellular Response to Virus’, ‘Cytokine Production’, and ‘Bacterial Defense Response’. This indicates that the endometrium is immune-suppressed. Immunosuppression heightens the risk of infection from pathogens.

The tissue damage caused by a bacterial infection will accelerate the endometrial tissue repair process. Therefore, innate immune genes are up-regulated in the endometrium of NEB dairy cows with respect to cell proliferation and differentiation. In addition, we found that the endometrium’s innate immune genes function to synthesize proteins. The mucosal immune system is a vital endometrial defensive mechanism. The mucus secreted by the mucous membrane contains proteins such as antimicrobial peptides and cytokines, which act as physical barriers and immune cells [62]. By inhibiting protein production and secretion, the endometrial mucosa’s immunological capacity and susceptibility to infection will be reduced. Due to the lower resistance of the peripheral mucosal tissue, the invasion of pathogenic bacteria and their products is increased, leading to an increase in the systemic inflammatory response. As shown in this study, the blood, liver, and hypothalamus also increased inflammatory, immune, or defense responses in the NEB group.

Meanwhile, it was found that NEB decreased humoral immune-related biological processes in the blood, as ‘Immunoglobulin Production’ and ‘B Cell Activation Involved in Immunological Response’ were drastically inhibited, signaling that the host was suffering from immunosuppression. Specific immunity is also reduced. Innate immune cells’ activity is promoted by systemic inflammation, as shown by the up-regulation of myeloid leukocyte-mediated immunity and myeloid leukocyte activation. Severe NEB is frequently accompanied by low blood glucose and high blood ketones. Hypoglycemia impairs the proper metabolism of blood cells, and high blood ketone, particularly BHBA, inhibits the function of innate immune cells, which is the main cause of immune suppression.

The liver is a key organ of the body’s host metabolism, and liver metabolic abnormalities can cause systemic metabolic illnesses. NEB affects dairy cow liver metabolism, cell differentiation, and sensitivity to outside stimuli. These mechanisms, especially lipid metabolism and substance transport, are up-regulated due to increased demand on liver lipid metabolism due to adipose mobilization during the NEB period and a heightened stress reaction to environmental perturbations. The liver is not only the major metabolic organ, but it is also a vital immune-related organ. It produces complement and vital cytokines. The stress reaction is most directly expressed by an increase in inflammatory responses, which are stimuli that disrupt the liver’s homeostasis. Due to energy shortage, the liver also reduces glucose metabolism and the calcium-mediated signaling pathway. Calcium signaling is typically a crucial activation signal for innate immunity, and the down-regulation of the calcium-mediated signaling response also indicates that several of the processes associated with innate immunity in the liver are similarly down-regulated.

The hypothalamus controls feeding, energy expenditure, and secretion. Energy restriction disrupts the hypothalamic-pituitary-ovarian axis, affecting cow ovulation [23]. The hypothalamus is also affected by NEB. In this work, we investigated the effects of NEB on the hypothalamus using the expression patterns of innate immunity genes and discovered that NEB cows have up-regulated inflammatory and immune-related biological processes and down-regulated substance transport and signal transduction pathways. The hypothalamus and adipose had up-regulated innate immunity pathways related to inflammation and immunity, while other tissues showed significant functional suppression. As a result, we suggest that the hypothalamus is another early tissue impacted by NEB. Material transport and signal transduction in the hypothalamus is inhibited, which is a key functional inhibition for the neurological system. Hypothalamic function inhibition may be an early sign of NEB deteriorating, as well as the primary reason for the development of subclinical and clinical ketosis. Therefore, a thorough investigation of NEB in dairy cows from multiple tissues is of considerable significance in showing the complicated relationship between metabolism and immunity in dairy cows throughout the perinatal period.

The expression pattern of 10 defense-related biological processes shared by different tissues in response to NEB (Figure 4C) showed that the endometrium had a decreased defense capacity, which facilitated pathogenic microorganisms to invade, while other tissues up-regulated relevant processes, which caused a systemic inflammatory response [63]. This is consistent with the findings that NEB causes systemic inflammation. In addition, systemic inflammation exhibited a greater effect on adipose tissue than on blood, liver, or hypothalamus.

To identify the genes that regulate metabolism and immunity in response to NEB, a list of innate immune genes that are differentially expressed across many tissues and have defensive roles was created. Innate immune genes PPI analysis of these differentially expressed genes revealed that IL-6 is the network hub linked with lipid metabolism and inflammation in dairy cows [12,20,64,65]. Among the proteins that interacted with IL-6, LBP, S100A12, ANXA1, LCN2, PTX3, IRF6, and CXCL2 were significantly differentially enhanced in a variety of tissues.

In response to NEB, LBP, PTX3, and S100A12 were significantly up-regulated in adipose, blood, and liver but down-regulated in endometrial. Dairy cow LBP mRNA and protein levels are linked to local and systemic inflammation [66,67]. The gene’s endometrial expression decreases the host’s ability to recognize infections and increases invasion risk. In response to inflammatory stimuli, PTX3 is activated in endothelial cells and mononuclear phagocytes by inflammatory cytokines. The protein controls inflammation and complement activation. Furthermore, it boosts angiogenesis and tissue remodeling. The protein is also a biomarker for many inflammatory diseases [68,69,70]. Gene expression overexpression enhances liver, blood, and adipose complement activation and systemic inflammation. Endometrial repair and immunological function are impaired by low gene expression [71,72]. S100A12 regulates inflammation and immunity. It recruits leukocytes, elevates cytokines and chemokines, and controls leukocyte adhesion and migration [73]. In cows, S100A12 is a marker for inflammatory responses and subclinical mastitis [74]. The upregulation of LBP, PTX3, and S100A12 in multiple tissues suggests that NEB influences systemic inflammation, and these three genes may serve as diagnostic indicators of systemic inflammation caused by NEB.CXCL2 regulates and inflames inflammation [75]. CXCL2 increased neutrophil adherence to visceral WAT endothelial cells, causing WAT inflammation [76]. LPS-treated rats have higher hypothalamic CXCL2 genes [77]. In this study, the increased expression of CXCL2 in adipose and brain tissues showed inflammation in the NEB group for both tissues. LCN2 is involved in hunger, energy metabolism, and adipose tissue inflammation and can pass the blood-brain barrier [78,79,80]. LCN2 increases glucose metabolism [78], reduces fat mass, and suppresses appetite. Appetite loss is a major contributor to the negative energy balance of perinatal cows, which drives adipose mobilization and the synthesis of ketone bodies to compensate for the increased glucose absorption by extrahepatic tissues [81]. LCN2, a key gene that links energy metabolism and immunity, could be employed as a molecular marker and marker-assisted selection for early diagnosis of negative energy balance. LBP, PTX3, S100A12, and LCN2 are upregulated in blood and tissues by NEB. These genes affect systemic inflammation and immunology. These genes may detect energy-induced systemic inflammation.

Notably, the gene most significantly overexpressed in the adipose tissue NEB group (log2FC = −10.4) is IRF6, which belongs to the interferon regulatory transcription factor (IRF) [82]. It is linked to liver damage [83] and obesity-related metabolic and immunological homeostasis [84,85]. In addition, increased expression of IRF6 in adipose and liver may be related to the inflammatory tissue damage caused by severe NEB [81]. Further research is needed to fully understand the effect of IRF6 in the NEB process.

However, the raw data came from a public database, and the different tissue samples did not come from the same individual. Because of this, the systematic analysis may not have been entirely accurate. Despite this, our research was able to provide light on the connection between a negative energy balance and systemic inflammation in dairy cows without causing any additional harm to the animals that were used in the experiment. To fully investigate the interactions between NEB and systemic inflammation, further research in the form of carefully designed studies is required.

## 5. Conclusions

The current study revealed that negative energy balance altered the expression pattern of innate immune genes in different tissues. By regulating biological processes related to immunity and metabolism, innate immune genes alter the host’s defense capacity and systemic inflammation. NEB can decrease the resistance of mucosal barrier systems such as the endometrium, raise the invasion rate of pathogenic microorganisms, activate the host systemic defense mechanism, and exacerbate systemic inflammation. The mRNA expression levels of LBP, PTX3, S100A12, and LCN2 were considerably altered in numerous tissues impacted by NEB and notably up-regulated in the blood. These genes are related to immunology and metabolism and have the potential to be exploited as diagnostic markers for NEB-related systemic inflammation and as screening markers for disease-resistant breeding. In addition, future research should comprehensively examine the role of IRF6 in the process of NEB in perinatal cows. This is a meaningful integrative transcriptome analyzed study to investigate the effects of NEB on defense and inflammation from the perspective of multiple tissues, providing new insights into the interaction between metabolism and immunity.

## Figures and Tables

**Table 1:** Detailed information about the RNA-seq data.

Trait Material	Data Grouping	Sequencing Platform	Data Sources	Accession Numbers
Adipose	Normal (PreP), *n* = 12 NEB (PP1), *n* = 12	Illumina NextSeq 500 (*Bos taurus*)	David Salcedo-Tacuma, 2020	GSE159224
Blood	Normal, *n* = 10 NEB, *n* = 13	Illumina HiSeq 2000 (*Bos taurus*)	Ze Yan, 2020	PRJNA605719
Endometrium	Normal (MNEB), *n* = 15 NEB (SNEB), *n* = 9	Illumina HiSeq 4000 (*Bos taurus*)	Wiruntita Chankeaw, 2021	GSE169638
Hypothalamus	Normal (Restricted), *n* = 14 NEB (high level), *n* = 7	Illumina HiSeq 4000 (*Bos taurus*)	Daragh Matthews, 2017	GSE49540
Liver	Normal (MNEB), *n* = 5 NEB (SNEB), *n* = 6	Illumina Genome Analyzer (*Bos taurus*)	Matthew McCabe, 2012	GSE37544

**Table 2:** The top 5 GO terms of up-regulated and down-regulated processes from every tissue.

GO Term	NES	Tissue	Regulated
Humoral Immune Response Mediated by Circulating Immunoglobulin	1.8095	Adipose	Up
Membrane Invagination	1.8004	Adipose	Up
Regulation of Humoral Immune Response	1.7919	Adipose	Up
Amyloid Beta Clearance	1.7888	Adipose	Up
Complement Activation	1.7642	Adipose	Up
Negative Regulation of Blood Vessel Diameter	−1.6923	Adipose	Down
Regulation of Steroid Biosynthetic Process	−1.702	Adipose	Down
Regulation of Steroid Metabolic Process	−1.7112	Adipose	Down
Fatty Acid Metabolic Process	−1.9598	Adipose	Down
Sterol Metabolic Process	−2.1124	Adipose	Down
Myeloid Leukocyte Mediated Immunity	2.2295	Blood	Up
Exocytosis	2.1093	Blood	Up
Myeloid Leukocyte Activation	1.9157	Blood	Up
Defense Response to Gram-Positive Bacterium	1.8822	Blood	Up
Cell Activation Involved in Immune Response	1.8773	Blood	Up
B Cell Activation	−1.9504	Blood	Down
Regulation of Fat Cell Differentiation	−2.0154	Blood	Down
Regulation of Immunoglobulin Production	−2.147	Blood	Down
B Cell Activation Involved in Immune Response	−2.2318	Blood	Down
Immunoglobulin Production	−2.2439	Blood	Down
Lymphocyte Chemotaxis	1.9792	Endometrium	Up
Protein-DNA Complex Subunit Organization	1.9613	Endometrium	Up
Organ Growth	1.949	Endometrium	Up
Heart Growth	1.8857	Endometrium	Up
Positive Regulation of Heart Growth	1.8108	Endometrium	Up
Cellular Response to Virus	−1.6434	Endometrium	Down
Positive Regulation of Response to Cytokine Stimulus	−1.6448	Endometrium	Down
Sterol Metabolic Process	−1.6475	Endometrium	Down
Regulation of Jun Kinase Activity	−1.6754	Endometrium	Down
Positive Regulation of Jun Kinase Activity	−1.7641	Endometrium	Down
Response to Retinoic Acid	1.9746	Hypothalamus	Up
Positive Regulation of CD4-Positive Alpha Beta T-Cell Differentiation	1.8528	Hypothalamus	Up
Regulation of CD4-Positive Alpha Beta T-Cell Differentiation	1.7604	Hypothalamus	Up
Leukocyte Homeostasis	1.6864	Hypothalamus	Up
Response to Chemokine	1.6836	Hypothalamus	Up
Non-canonical Wnt Signaling Pathway	−1.6246	Hypothalamus	Down
Calcium Ion Transmembrane Import into Cytosol	−1.6279	Hypothalamus	Down
Neuron Projection Organization	−1.6319	Hypothalamus	Down
Cellular Response to Ketone	−1.6627	Hypothalamus	Down
Regulation of Calcium Ion Transmembrane Transport	−1.6661	Hypothalamus	Down
Tissue Migration	1.695	Liver	Up
Negative Regulation of Cell Adhesion	1.6623	Liver	Up
Negative Regulation of Coagulation	1.6527	Liver	Up
Odontogenesis	1.65	Liver	Up
Positive Regulation of Cell Division	1.6472	Liver	Up
Calcineurin-Mediated Signaling	−1.656	Liver	Down
Positive Regulation of Lipid Kinase Activity	−1.6596	Liver	Down
Glucose Metabolic Process	−1.6671	Liver	Down
Establishment of Cell Polarity	−1.7294	Liver	Down
Positive Regulation of Phospholipid Metabolic Process	−1.7409	Liver	Down

## Data Availability

The data presented in this study are available either within the article or as Supplementary Materials.

## Supplementary Materials

The following supporting information can be downloaded at: https://www.mdpi.com/article/10.3390/vetsci10020107/s1, Table S1: List of tissue-specific highly expressed innate immune genes; Table S2: DEGs of innate immune genes from multi-tissues.
